# Supplementary material for: Characteristics and etiologies of hepatocellular carcinoma in patients without cirrhosis: When East meets West
Source: PLoS One. 2021 Jan 13;16(1):e0244939. doi: 10.1371/journal.pone.0244939 (PMC7806135; doi:10.1371/journal.pone.0244939)
Supplement: Attachment — Submitted filename: response to Reviewer r2.docx [file pone.0244939.s002.docx]

Reviewer #1: Characteristics and etiologies of hepatocellular carcinoma in patients without cirrhosis: when East meets West

Yen et al.

Yen et al. have performed a retrospective review of patients in their center’s HCC registry database and identified patients who presented with HCC in the presence vs. absence of cirrhosis, with the goal of identifying patient characteristics and outcomes associated with non-cirrhotic HCC. This study includes a large sample (529 total patients) who presented with HCC in the absence of cirrhosis. There are certain elements of the manuscript that require further elaboration and clarification. The strengths of the cohort include accurate treatment and survival data given the Taiwanese healthcare system.

Major issues/comments:

1) This reviewer has several comments/questions related to the criteria for “no cirrhosis”, specifically:

a. Please provide the specific diagnostic criteria for defining the presence vs. absence of cirrhosis on imaging. For the patients who had histologic diagnosis available, did the findings on histology correlate with the findings on imaging? How was evidence of portal hypertension adjudicated?

**Response:** Thank you so much for your comments.

1. The imaging features of cirrhosis are as follows: nodular liver surface, blunt edge, small liver size with relative enlargement of the caudate lobe or left lobe, coarse or heterogeneous liver parenchyma, and regenerative nodules [12]. Please see page 7, red color paragraph. In our clinical practice, however, there are no specific diagnostic criteria for defining the presence vs. absence of cirrhosis on imaging. Relatedly, the diagnosis of cirrhosis on imaging was subjective in the present study. please see page 26, line 2-4.
2. Diagnostic accuracy of imaging for cirrhosis:

We randomly selected 202 patients who had undergone surgical resection to evaluate the correlation between imaging findings and histology for cirrhosis. We reviewed the CT reports or MRI reports (if the former is not available) of these patients. Among 202 patients, 20 patients had alcohol use disorder, 112 patients were HBsAg positive, 52 patients were anti-HCV positive, 131 patients were diagnosed as non-cirrhotic by imaging (109 patients were histologically non-cirrhotic on histology, 22 patients were histologically cirrhotic), 71 patients were diagnosed as cirrhotic by imaging (30 patients were histologically non-cirrhotic, 41 patients were histologically cirrhotic). Using histology as a reference, the imaging diagnostic accuracy, sensitivity, specificity, positive predictive value, and negative predictive value for cirrhosis were 74.3%, 65.1%, 78.4 %, 57.7 %, and 83.2 %, respectively. Please see page 19, first paragraph

3. Signs of portal hypertension on imaging studies such as ascites, splenomegaly, and portosystemic collateral vessels (doi: 10.1007/s12072-016-9760-3) were not recorded in our HCC registry data. In our clinical practice, signs of portal hypertension on imaging studies increase the confidence in the diagnosis of cirrhosis.

b. In the patients who had the presence or absence of cirrhosis assessed by imaging (but not histology), how many had ultrasound vs. CT scan vs. MRI?

**Response:** Thank you so much for your comments.

This data was not recorded in our HCC registry data. In our clinical practice, however, ultrasound is performed in all patients with HCC. Contrast-enhanced CT or MRI is performed, meanwhile, in patients with HCC and adequate renal function (i.e. estimated glomerular filtration rate ≥30 ml/min/1.73 m^2^), whereas non-contrast-enhanced MRI is performed in patients with HCC and severe renal impairment (i.e. estimated glomerular filtration rate <30 ml/min/1.73 m^2^).

In the discussion section, the authors comment on the sensitivity and specificity of ultrasound in diagnosing cirrhosis, which leads this reviewer to believe that most patients in this cohort who had an imaging diagnosis of cirrhosis vs. no cirrhosis had this diagnosis made by ultrasound. To perform TNM staging for patients with HCC, evaluation for metastatic disease typically requires cross-sectional imaging such as CT or MRI. If the majority of patients therefore had cross-sectional imaging (and not only ultrasound), this information about ultrasound specificity and sensitivity for cirrhosis in the authors’ previous study does not seem relevant.

**Response:** Thank you so much for your comments.

We have deleted the information about ultrasound specificity and sensitivity for cirrhosis from our previous study.

2) The second major concern of this reviewer is the lack of data on patients without HBV, HCV, or alcohol-related liver disease. This reviewer believes that this is a major limitation and warrants acknowledgement, as 17.8% of patients have an unknown underlying etiology of liver disease. The statement that only 4.6% of patients in the entire cohort might have NAFLD-associated, non-cirrhotic HCC minimizes this limitation.

a. This reviewer asks the authors to consider including this as a study limitation.

b. Are specific patient characteristics known for those with unknown etiology of liver disease? I.e. do these patients have increased rates of diabetes, hypertension, dyslipidemia, obesity or other risk factors we would typically associate with NAFLD?

**Response:** Thank you so much for your comments.

1. We have now mentioned the unknown underlying etiology of liver disease in patients without HBV, HCV, or alcohol-related liver disease as a study limitation, as follows: “There were also some limitations in the present study … Third, there was a lack of etiology data on patients without HBV, HCV, or alcohol-related liver disease (such as NAFLD).”please see page 26
2. Diabetes, hypertension, dyslipidemia, and other risk factors typically associated with NAFLD (e.g. metabolic syndrome, truncal obesity defined by waist circumference) were not recorded in our HCC registry data. However, the HCC registry data of our institution do include BMI data. Obesity is defined as BMI ≥27(kg/m2) by the Health Promotion Administration, Ministry of Health and Welfare, Taiwan (https://health99.hpa.gov.tw/onlinkhealth/onlink_bmi.aspx). Among those with HBV, HCV, or alcohol-related liver disease, there were 443 (25.29%) patients with BMI ≥27(kg/m2). Among those with unknown etiology of liver disease, there were 95 (31.35%) patients with BMI ≥27(kg/m2). The proportion of patients with BMI ≥27 (kg/m2) was, therefore, significantly higher among those with unknown etiology of liver disease compared with those with HBV, HCV, or alcohol-related liver disease (p=0.002). please see page 26

Minor issues/comments:

1) For those patients with histologic diagnosis of no cirrhosis – is there more detailed information on the stage of fibrosis other than “no cirrhosis”? Are patients without cirrhosis but higher stages of fibrosis, for example, more at risk of developing non-cirrhotic HCC than the patients without cirrhosis and lower stages of fibrosis? Were any noninvasive measurements (FIB-4, NAFLD-FS, APRI) calculated to help estimate numbers?

**Response:** Thank you so much for your comments.

1. Among 670 patients, 87 patients had an Ishak fibrosis score of 5, and 234 patients had an Ishak fibrosis score of 6. The remaining 349 patients were non-cirrhotic. Among the 349 non-cirrhotic patients, 169 (48.4%) patients had lower stages of fibrosis (i.e. Ishak fibrosis scores of 0-2, which represent the absence of bridging fibrosis), and 180 (51.6%) patients had higher stages of fibrosis (i.e. Ishak fibrosis scores of 3-4, which represent the presence of bridging fibrosis) [10]. Please see page 9 and 10, red color paragraph.
2. The HCC registry data for our institution do not include AST, ALT, platelet count, fasting glucose, diabetes, or albumin levels. Relatedly, noninvasive measurements (FIB-4, NAFLD-FS, APRI) were not available in the present study.

2) Do the authors have information on patient medical comorbidities? Are there certain comorbidities with associated increased rates of non-cirrhotic HCC?

**Response:** Thank you so much for your comments.

The HCC registry data for our institution do not include patient medical comorbidities. We have including this as a study limitation. Please see page 27, line 5.

3) Please define “habitual drinking” by standards associated with diagnostic criteria.

**Response:** Thank you so much for your comments.

Drinking behavior is recorded in our HCC registry data as follows:

the 000 code refers to someone who has never had an alcoholic drink in their life, while the 001 code refers to someone who may have drank alcohol in the past but is currently abstinent. Code 002 indicates occasional drinking. Code 003 indicates habitual drinking for more than 10 years. Code 004 indicates habitual drinking for less than or equal to 10 years. Code 009 indicates habitual drinking for unknown number of years. The HCC registry data for our institution do not, however, include daily alcohol intake or data provided by the use of screening tools [e.g. the AUDIT (Alcohol Use Disorders Inventory Test)] to identify alcohol use disorders [29]. We have including this as a study limitation. Please see page 27, first paragraph
